# Navigating the dementia caregiving journey: A scoping review protocol of interventions and their responsiveness to caregivers’ evolving needs across the illness trajectory

**DOI:** 10.1371/journal.pone.0333475

**Published:** 2025-09-30

**Authors:** Kristina M. Kokorelias, Nathan M. Stall, Marina Wasilewski, Monique A.M. Gignac, Nira Rittenberg, Hardeep Singh, Erica Dove, Maurita Harris, Ron Beleno, Gary Naglie, Jill I. Cameron

**Affiliations:** 1 Division of Geriatric Medicine, Department of Medicine, Sinai Health System and University Health Network, Toronto, Ontario, Canada; 2 Department of Occupational Science & Occupational Therapy, Temerty Faculty of Medicine, University of Toronto, Toronto, Ontario, Canada; 3 National Institute on Ageing, Toronto Metropolitan University, Toronto, Ontario, Canada; 4 Rehabilitation Sciences Institute, Temerty Faculty of Medicine, University of Toronto, Toronto, Ontario, Canada; 5 Institute of Health Policy, Management and Evaluation, University of Toronto, Toronto, Ontario, Canada; 6 St. John’s Rehab Research Program, Sunnybrook Health Sciences Centre, Toronto, Ontario, Canada; 7 Institute for Work & Health, Toronto, Ontario, Canada; 8 Dalla Lana School of Public Health, University of Toronto, Toronto, Ontario, Canada; 9 Faculty of Liberal Arts, Wilfrid Laurier University, Brantford, Canada; 10 Department of Medicine and Rotman Research Institute, Baycrest Health Sciences, Toronto, Ontario, Canada; Erasmus University Rotterdam, NETHERLANDS, KINGDOM OF THE

## Abstract

**Introduction:**

Family caregivers of persons with dementia (PWD) provide critical and often sustained support across the dementia trajectory. Despite growing recognition of their evolving needs, many interventions remain episodic and not tailored to different caregiving phases. Frameworks like the Timing It Right (TIR) and the Caregiver-Identified Phases of Alzheimer’s Disease (CIP-AD) offer structured approaches to understanding how caregiver needs change over time. However, little is known about whether current interventions align with these phase-specific needs.

**Objectives:**

This scoping review aims to (1) explore the extent to which existing dementia caregiving interventions address different stages of the caregiving journey or dementia progression; (2) summarize and characterize these interventions using the Template for Intervention Description and Replication (TIDieR) checklist; and (3) identify characteristics of the populations targeted by these interventions.

**Methods:**

Following JBI methodology and the PRISMA-ScR checklist, we will conduct a scoping review of randomized controlled trials, non-randomized controlled trials, and quasi-experimental studies focused on interventions for unpaid family caregivers of community-dwelling PWD. Studies will be included if they address at least one phase of the dementia trajectory and are delivered in community-based settings. A comprehensive search of six databases from 1995 to 2025 will be developed and peer-reviewed using the PRESS framework. Data will be extracted using a standardized form and analyzed thematically.

**Significance:**

This review will map how dementia caregiving interventions address the evolving needs of caregivers over time and inform future development and implementation of phase-responsive support strategies. The findings will guide research, policy, and practice in creating caregiver-centered interventions that reflect the realities of caregiving across the dementia continuum.

## Introduction

Dementia is a progressive, degenerative condition that not only impacts individuals but also places a substantial burden on family members and friends who often provide considerable care (unpaid family caregivers) [1]. As the disease progresses, family caregivers play an essential role in supporting persons with dementia (PWD), and in navigating complex medical, social, and emotional challenges [2]. These family caregivers are often the primary providers of care, managing daily activities, medical needs, and decision-making, which can be physically, mentally, and emotionally taxing for family caregivers [3]. Thus, the role of family caregiving is integral to dementia care, influencing the quality of life for both the individual with dementia and the caregivers themselves.

Despite their critical role, family caregivers to PWD a often lack adequate support in navigating the complexities of dementia care [4]. For instance, many caregivers struggle to access timely respite care or coordinate healthcare services due to fragmented systems [5,6]. While a range of programs and services exist to assist family caregivers, including education, respite care, and psychosocial interventions, many family caregivers to persons with dementia underutilize these resources [7,8]. Importantly, while many interventions exist, the evidence base supporting widespread funding and dissemination of most models remains scrace. Only a few interventions, such as collaborative care models and REACH II (and its adaptations), have demonstrated consistent effectiveness [9]. Barriers such as lack of awareness, time constraints, stigma, and difficulty accessing services contribute to family caregivers low uptake of available supports [8,10]. As a result, many family caregivers manage their responsibilities with limited external assistance, increasing their risk of burnout. Ensuring that services are both accessible and responsive to family caregivers’ evolving needs is essential in sustaining their well-being and ability to provide care over time [11].

Also important to consider is that family caregivers to persons with dementia have needs that are not static and that evolve in response to the changing nature of the disease [11,12], as well as changing life circumstances including caregiver health [13]. As the dementia caregiving journey progresses [14], caregivers may experience increased stress, burnout, and isolation [15]. Research shows that these evolving and complex needs are often not sufficiently addressed by existing systems of care [16–18].

To address unmet needs, recent efforts have aimed to better meet the needs of caregivers to persons with dementia [19,20]. For example, programs such as Dementia Friendly Communities initiatives, aim to better support family caregivers within their communities and often incorporate family caregivers into care planning and decision-making processes, ensuring they have access to relevant information and support [21]. At the systems level, the *Guiding an Improved Dementia Experience (GUIDE) Model*, launched by the Centers for Medicare & Medicaid Services (CMS) in 2024, represents a major step forward. This eight-year, nationwide model aims to deliver comprehensive, coordinated dementia care, including care navigation, caregiver training, 24/7 support lines, respite services, and linkages to community supports, through a standardized approach and alternative payment structures [14].

Similarly, initiatives like REACH II (Resources for Enhancing Alzheimer’s Caregiver Health) offer structured training to help family caregivers develop skills in dementia management, communication, and self-care [22]. However, while there has been recognition of the importance of supporting caregivers throughout the dementia disease trajectory, the focus has typically been on episodic interventions, often failing to account for the nuanced, phase-specific needs that arise as the disease trajectory progresses [11]. On the contrary, the GUIDE Model is particularly notable for its strong caregiver component [14]. It requires participating organizations to support unpaid caregivers with evidence-based education, psychosocial resources, and respite care, addressing the emotional and physical toll of caregiving [14]. The model also incorporates health-related social needs screening and prioritizes equity by adjusting funding for underserved populations [14].

Frameworks have emerged to structure the involvement of caregivers in care decisions to increase our understanding of changes in the caregiving situation across disease trajectory and across family caregivers’ corresponding need for support. One such framework for caregivers to persons with stroke, the Timing It Right (TIR) framework, emphasizes the importance of delivering interventions at the right time, recognizing that caregivers’ needs change as the disease progresses [12]. The TIR framework outlines five phases that examine experiences and support needs of family caregivers to persons with stroke: 1) the illness event/diagnosis; 2) period of stabilization; 3) preparation for discharge from hospital or inpatient rehabilitation; 4) first few months of home adjustment (implementation), and 5) longer-term adjustment to community living (adaptation) [12]. This framework was instrumental in guiding the development of the Caregiver-Identified Phases of Alzheimer’s Disease (CIP-AD) framework that extends the notion of time-based caregiver support to the context of caregiving for persons with dementia by outlining five distinct phases of caregiving across the Alzheimer’s Disease trajectory, each with its own set of challenges and support needs [11]. Thus, the CIP-AD framework aims to provide a roadmap for identifying and addressing caregivers’ specific needs at various stages of dementia, ensuring that interventions are responsive to the changing dynamics of caregiving [11]. The central premise of these frameworks is that careful attention to phase-specific needs will enhance family caregiver ability to receive support and sustain the caregiving role.

While these frameworks provide a structured approach to understanding and supporting family caregivers, there remains a gap in how well existing interventions align with caregivers’ evolving needs across the dementia trajectory. Without a comprehensive understanding of how well current interventions correspond to phase-specific needs, family caregivers for persons with dementia may continue to face unmet needs and challenges related to critical transitions and changing circumstances, including the transition to long-term care. A review of existing interventions can assess whether existing interventions are aimed at addressing caregivers’ evolving needs, when and how they are delivered, and whether they change in response to the demands of different caregiving phases.

We outline a scoping review protocol that seeks to better understand the scope of the existing literature on dementia caregiving interventions in the context of the evolving needs of family caregivers.

## Objectives

In this scoping review, we aim to 1) Explore the existing literature on dementia caregiving interventions to to determine which stages of the caregiving journey or dementia progression the interventions address and analyze the methods and approaches used to deliver support to caregivers, including the format, frequency, and mode of intervention delivery; 2) summarize and characterize interventions based on the components identified within the Template for Intervention Description and Replication (TIDieR) checklist [23]; and 3) identify the characteristics of the populations targeted by studies of interventions for dementia caregivers.

## Methods

We will carry out this scoping review in accordance with the JBI Scoping Review methodology [24,25]. Our protocol was developed using the Preferred Reporting Items for Systematic Review and Meta-Analysis Protocols (PRISMA-P) (S1 File) [26]. We will follow the JBI methodology for scoping reviews and adhere to the PRISMA-ScR checklist for reporting findings [27]. Additionally, the review protocol has been registered on the Open Science Framework [osf.io/7hae8]. A scoping review is appropriate for this study as it allows for a comprehensive examination of the breadth and nature of existing dementia caregiving interventions, mapping how they address caregivers’ evolving needs across the illness (syndrome) trajectory [28].

While the protocol is registered on the Open Science Framework, its publication in this journal adds value by providing a detailed methodological blueprint for assessing how interventions align with the evolving, phase-specific needs of family caregivers to persons living with dementia, offering a level of methodological transparency and applicability beyond that provided in a registry entry alone. Publication of this protocol in a peer-reviewed journal ensures methodological transparency and allows for critical appraisal by the field, promoting uptake and informing future research, policy, and intervention design. This is particularly important given the evolving nature of caregiver needs across the dementia trajectory, which requires interventions to be responsive and timely.

Our scoping review will include engagement with knowledge users, including caregivers of persons with dementia, healthcare providers, and representatives from organizations that support dementia caregivers, through an integrated knowledge translation (iKT) panel in collaboration with the research team [29]. Knowledge users have been recruited through the research team’s established relationships with key knowledge users, including leading organizations in dementia care. They will provide valuable input on search terms to enhance the depth and scope of our search strategy, contribute to the article screening process, and be involved throughout the interpretation and dissemination phases of the project.

### Eligibility criteria

#### Participants.

This review will include studies focusing on caregivers of community-dwelling persons living with Alzheimer’s Disease and Related Dementias (ADRD) (e.g., Alzheimer’s Disease, Vascular Dementia, Lewy Body Dementia, Frontotemporal Dementia, Mixed Dementia, Parkinson’s Disease Dementia, Creutzfeldt-Jakob Disease, Huntington’s Disease, Normal Pressure Hydrocephalus). Caregivers are defined as family members, friends, or other unpaid individuals who provide physical, emotional, and social support to persons with dementia across various stages of the disease trajectory [30]. These may include spouses, adult children, siblings, or other relatives who take on caregiving responsibilities, whether they reside with the person with dementia or provide care from a distance.

We will exclude studies if they focus on family caregivers of individuals with other cognitive or neurological disorders, as these populations may not have the same caregiving needs or challenges as those caring for individuals with dementia. We will also exclude caregivers caring for persons living in nursing homes and long-term care. Additionally, interventions targeting professional caregivers or paid staff, rather than unpaid caregivers, will be excluded. The review will also exclude research on interventions that are not specifically designed for dementia caregiving, such as general family caregiving interventions that are not tailored to the unique needs of dementia caregivers. For studies with mixed caregiver samples, we will include those where more than half of the participants are caregivers to persons with dementia, to ensure relevance to the dementia caregiving context.

Interventions that simultaneously target both the person with dementia and their family caregiver (i.e., dyadic interventions such as the GUIDE Model [14]) will be included if they contain a component explicitly designed to address the needs of family caregivers.

#### Concept.

The review will examine interventions designed to support family caregivers of persons with dementia. For the purposes of this review, ‘interventions’ are defined broadly to include structured, purposeful non-pharmacologic strategies, programs, or services intended to improve caregiver well-being, enhance caregiving skills, or reduce caregiver burden. Based on major evidence syntheses, representative intervention categories included psychoeducation, skill-building/training, counseling and psychotherapy, respite services, peer-support, care navigation and case management, dyadic programs, technology-supported interventions, and multicomponent or collaborative-care models. These categories were informed by recent large-scale evidence reviews of non-drug care interventions for persons with dementia and their caregivers. [31] We will only include randomized controlled trials, non-randomized controlled trials and quasi-experimental designs, to be consistent with an existing large review on evaluating the overall effectiveness of a wide range of non-drug care interventions for PWD and their formal and family caregivers.

Studies must specifically evaluate interventions that address at least one stage of dementia (e.g., early, moderate, or advanced) or a specific phase of the caregiving journey (e.g., support during diagnosis, assistance with activities of daily living). Eligible studies should examine when and how interventions are delivered, as well as their adaptability to changing caregiving demands over time (if at all).

For the purposes of this review, we treat the term “delivery” as encompassing both how an intervention was provided to caregivers and the implementation features that supported that delivery.

#### Context.

Studies will be included if they take place in settings where community-dwelling family caregiving for dementia occurs, such as community-based settings and home care. For the purposes of this review, we define “community-dwelling family caregiving” broadly to include care provided in private homes and apartments and supports delivered in community settings. Examples include in-home care and visiting nursing; primary care and outpatient clinics where caregiver support is offered; adult day programs and community centres that provide social and health-related services; faith-based and volunteer programs; community mental health, palliative, and hospice services delivered in the community; formal respite and short-stay programs; meal delivery and transportation services; and telehealth, digital, and care-navigation supports. We will exclude inpatient facilities and long-term care settings. Research will not be limited to any specific healthcare system, allowing for comparisons across diverse geographic and socioeconomic contexts. We will include articles describing studies conducted anywhere in the world will be included, ensuring a broad understanding of dementia caregiving interventions across various cultural and healthcare environments.

To align with the historical development of dementia caregiving research, the review will include studies published from January 1995 to the present. This timeframe is chosen based on the establishment of the Resources for Enhancing Alzheimer’s Caregiver Health (REACH) program in 1995, which was one of the first large-scale, multi-site research initiatives focused on interventions to support family caregivers of individuals living with Alzheimer’s disease and related dementias [32].

Peer-reviewed articles in languages other than English will be considered, provided they are either originally written in English or translated into English using tools such as Google Translate [33].

### Search strategy and information sources

We will develop a comprehensive search strategy with our information specialist to identify relevant peer-reviewed studies. The search will be conducted in MEDLINE (Ovid) and Medline (Ovid) Epub Ahead of Print, In-Process and Other Non-Indexed Citations, CINAHL (EBSCO), AgeLine (EBSCO), EMBASE (Ovid) databases and PsycINFO (Ovid) from 1995-August 14 day 2025. No language limits will be applied.

In addition to the search strategy development, McGowan and colleagues established evidence-based guidelines for the Peer Review of Electronic Search Strategies (PRESS) in systematic reviews and other forms of evidence synthesis [34]. This will be done by having the primary search conducted by an experienced librarian, followed by a review of the search strategy by a second librarian to examine its comprehensiveness and enhance the search if necessary. This process will adhere to the PRESS guidelines to maintain rigour and reliability.

Search terms will include a combination of keywords and subject headings related to dementia caregiving, caregiver interventions, caregiving needs across the dementia trajectory, and the specific phases of caregiving.

### Study/Source of evidence selection

After conducting the search, we will import all identified references into Covidence (Veritas health innovation, Melbourne, Australia), where duplicates will be removed [35]. We will carry out a pilot test of the Level 1 screening form, based on the eligibility criteria outlined earlier, will be carried out on a random sample of 25% of the identified articles. The search terms and inclusion/exclusion criteria will be applied systematically, and the consistency of study selection will be assessed using Cohen’s kappa score [36]. If a low agreement rate (i.e., < 70% [37]) is observed between reviewers, the descriptions of the eligibility criteria will be revised and clarified to ensure consistent application of the selection process. The Level 1 and Level 2 screening processes will be performed independently by two reviewers. For Level 1 screening, reviewers will assess the titles and abstracts of the identified articles to determine inclusion based on the screening form. For Level 2 screening, full-text articles of potentially relevant studies will be collected and reviewed to determine final inclusion. A pilot test of the Level 2 screening form will also be conducted on 25% of the articles, followed by the calculation of inter-rater reliability using Cohen’s kappa score. If the kappa score is found to be below 0.70, indicating low agreement between reviewers, the eligibility criteria will be revised and clarified to ensure consistent application of the selection process [37]. After revisions, the pilot test will be re-assessed to confirm improved consistency before proceeding with full screening. We will resolve any discrepancies during the screening process will be resolved through discussion between the two reviewers. If needed, a third reviewer (senior author) will be consulted to resolve any conflicts. For full-text screening, all studies excluded during the screening phases will be documented in Covidence, along with the rationale for their exclusion.

### Data items and charting process

We will extract data from the literature using a standardized abstraction form developed on Covidence. The data abstraction form will be pilot tested and refined by at least two members of the research team. Pilot testing will involve testing the form on a subset of studies to ensure that it is user-friendly and effectively captures the relevant data consistently across all studies. During this process, any issues such as unclear instructions, missing categories, or ambiguities will be identified and addressed. The form will then be revised to improve clarity and accuracy in data collection. Additionally, this refinement process will allow for adjustments based on the specific characteristics of the studies included in the review, ensuring the abstraction form is appropriate for the full set of studies.

We will perform data abstraction independently by two reviewers to ensure accuracy and consistency. The data abstraction process will be directly aligned with the three aims of this scoping review, ensuring that relevant data is captured for each objective. First, study characteristics will be documented, including the study design, location (e.g., whether the intervention took place in home care, long-term care, or community settings), and sample size, to provide context for the research and ensure its relevance to the review. This will help to identify trends or patterns across different study types and settings.

We will extract data on each intervention. Specifically, we will investigate if and how these interventions adapt over time, adjusting to the shifting responsibilities and emotional needs of caregivers as they navigate the different phases of dementia caregiving (Aim 1). We will examine the timing and frequency of the interventions to determine whether they are tailored to the evolving challenges caregivers face as the disease progresses. This could include changes in intervention content, delivery methods (e.g., in-person sessions transitioning to online support), or the involvement of different types of support providers (e.g., healthcare professionals, peer caregivers, family members). In line with the second aim, we will also extract other relevant components related to the intervention. These elements include the intervention’s name, rationale, and aims; the materials used (e.g., printed materials, online resources); the procedures involved (e.g., delivery methods and strategies); who delivers the intervention (i.e., provider characteristics, such as healthcare professionals, peer support, or family members); the frequency and duration of the intervention (e.g., one-time versus ongoing support, number of sessions, session length); the setting in which the intervention occurs (e.g., home care, hospital, community center) [23]; and the implementation of the intervention.

To identify the characteristics of the populations targeted by studies of dementia caregiving interventions (Aim 3), data will be abstracted on the caregiver and care recipient sociodemographic and clinical characteristics. Data abstraction will be informed by the Sex and Gender Plus framework, which emphasizes not only sex and gender but also intersecting social identities [38]. For caregivers, this will include age, sex assigned at birth, gender identity (e.g., male, female, non-binary), sexual orientation, cultural background, socioeconomic status, educational background, and caregiving experience. In addition, data will be collected about the persons with dementia being cared for, with attention to their age, type of dementia (e.g., Alzheimer’s disease, vascular dementia, Lewy body dementia), stage of disease, comorbidities, medications (including psychotropic medications such as antipsychotics and sedatives), presence of responsive behaviours/behavioural and psychological symptoms of dementia, gender identity, cultural background, and relationship to the caregiver.

We will code and analyze interventions that simultaneously target both the PWD and their family caregiver as “dyadic caregiving interventions” to differentiate them from caregiver-only or PWD-only interventions. Data extraction will include details on how caregiver-specific needs are addressed within the broader intervention only.

While the primary focus will be on the characteristics and components of the interventions, we will conduct a critical appraisal of the included RCTs to assess their methodological quality. This appraisal will involve evaluating factors such as randomization methods, blinding, sample size, and potential biases, using established appraisal tools such as the Cochrane Risk of Bias tool [39]. The purpose of this critical appraisal is not to exclude studies based on their quality but to understand the strength of the evidence and the potential risk of bias.

### Data analysis and synthesis

We will follow a systematic approach to consolidate and interpret the findings from the included studies, in line with JBI guidelines [40]. The review will engage in data analysis and presentation through: (1) providing a descriptive numerical summary of the included studies and conducting a deductive content analysis, (2) reporting findings in relation to the study objectives, and (3) discussing the overall implications of the results for future dementia caregiving interventions [40]. Given the nature of the scoping review, the primary objective will be to map the existing literature, identify trends, and provide an overview of the types of interventions, their delivery, and how many have the potential to address the evolving needs of dementia caregivers. This will be done using a narrative synthesis. The synthesis will not involve a formal meta-analysis, as the studies are expected to be diverse in terms of design, intervention type, and outcomes.

Initially, we will use descriptive statistics to summarize the key characteristics of the included studies, such as study design, participant demographics, intervention types, and outcome measures. Frequencies and percentages will be used to summarize how many interventions target specific dementia stages (early, moderate, advanced), caregiving phases (e.g., assistance with diagnosis, activities of daily living), and intervention components (e.g., delivery format, duration, mode of support). This will allow for a broad understanding of the landscape of dementia caregiving interventions and help identify any gaps in the literature.

An inductive and deductive content analysis will be applied to synthesize qualitative data extracted from the included studies [41]. This analysis will align with the review’s three aims: (1) mapping the characteristics of interventions, including those that could address caregivers evolving needs, (2) summarizing intervention components using the TIDieR framework [23], and (3) identifying the populations targeted by the interventions, using the Sex and Gender Plus framework to capture demographic and equity-related factors [38]. The extracted data will be imported into NVivo 14 for organization and coding [42]. Two reviewers will independently familiarize themselves with the data by reading and re-reading the extracted information to identify patterns and ensure a thorough understanding. Any discrepancies in coding will be resolved through discussion, with input from a third reviewer if needed. A deductive coding approach will be used to code intervention components according to the TIDieR framework. In addition to the predefined categories, an inductive coding process will be applied to identify emerging findings that may not be captured by existing frameworks. This will include an analysis of how interventions evolve over time to meet changing caregiver needs, as well as barriers and facilitators to intervention uptake. NVivo will be used to generate word clouds, frequency counts, and relationships to support visualization and interpretation [42].

We will present the results as a narrative synthesis, that will provide an overview of trends in the intervention literature and discuss gaps in research, particularly in relation to whether and how interventions adapt to caregivers’ evolving needs across the dementia trajectory. Findings will also be contextualized within broader discussions of dementia caregiving support, informing future research and policy directions.

### Presentation of results

We anticipate results in 2026. The results section of the scoping review will consist of two main parts: the first detailing the outcomes of the search strategy and selection process, including a PRISMA flow diagram, and the second highlighting the key information or results relevant to the review’s objectives or questions [27]. The results will be displayed in a tabular format, to organize the data extracted from the included studies. Next, results will be presented descriptively in alignment with the review’s objectives and scope.

### Patient and public consultation

Levac et al. emphasize that incorporating the optional consultation step outlined by Arksey and O’Malley enhances methodological rigour [28]. Our research team engaged with caregivers of persons with dementia, as well as representatives from community organizations supporting caregivers, including two Alzheimer’s Societies in Canada (i.e., Alzheimer Society of Toronto and Alzheimer Society of Peel). This group of interest holders played a key role in refining our research questions and tailoring our review objectives to better align with their needs and priorities.

Throughout the review process, we will continue to engage these interest holders, not as active reviewers, but as advisors at each key stage of the study. Their role will involve providing feedback on the inclusion and exclusion criteria, ensuring that the interventions reviewed are meaningful to real-world caregiving experiences, and advising on the categorization and interpretation of findings. They will also review the data abstraction framework with the research team to ensure that the extracted information accurately captures aspects most relevant to caregivers.

As preliminary findings emerge, they will be shared with this group for discussion, allowing them to provide insights on the significance of the results and their implications for future dementia caregiving interventions. This input will help contextualize the findings and identify key areas where interventions could be strengthened or adapted to better support caregivers throughout the dementia trajectory. By incorporating the perspectives of caregivers and community organizations, we aim to ensure that the review has practical value and contributes to the development of interventions that are both evidence-based and responsive to caregivers’ evolving needs.

### Dissemination

We expect results from our review in early 2026 and will employ a multifaceted dissemination strategy will be employed to maximize the impact of our findings among both academic and stakeholder audiences. The findings will be submitted for publication in a peer-reviewed academic journal, targeting high-impact journals in gerontology, dementia care, and health services research, such as The Gerontologist, Aging & Mental Health, or Journal of the American Geriatrics Society. Additionally, we will present our results at national and international academic conferences, with potential venues including the Canadian Association on Gerontology (CAG) Annual Scientific and Educational Meeting, the Alzheimer’s Association International Conference (AAIC), the International Conference on Integrated Care (ICIC), Canadian Geriatrics Society (CGS) and the American Geriatrics Society (AGS) Annual Meeting.

Beyond traditional academic dissemination, we will engage in knowledge translation activities tailored to interest holder groups, ensuring that our findings are accessible and actionable for those working directly with caregivers. We will present findings at academic rounds and research seminars at the investigators’ affiliated universities, such as the University of Toronto, as well as hospital-based research institutes specializing in geriatrics, dementia care, and caregiving.

To reach frontline care providers, policymakers, and community organizations, we will share our results through tailored knowledge translation presentations to our stakeholder partners, including Alzheimer’s Societies across Canada, caregiver advocacy groups, and organizations supporting persons with dementia. This may include webinars, virtual town halls, and briefings designed to facilitate discussion on how the findings can be incorporated into practice and policy.

Additionally, we will develop plain-language summaries, infographics, and policy briefs, which will be disseminated through organizational newsletters, social media platforms, and caregiver support networks to ensure accessibility for caregivers and the broader public. We will also explore collaborations with stakeholders to create practical tools, such as decision aids or training modules, based on our findings, to support caregivers and healthcare providers in navigating dementia caregiving interventions effectively.

## Discussion

The aim of the forthcoming review is to provide a synthesis of the scope and content of existing dementia caregiving interventions and assess their alignment with the evolving needs of family caregivers across the illness trajectory. Unlike the AHRQ review, which broadly evaluates the effectiveness of dementia care interventions up to 2020, we focus on synthesizing the scope and content of existing caregiving interventions and critically examining how well they align with the evolving and often under-addressed needs of family caregivers across the full dementia illness trajectory [20]. Specifically, this review will examine how interventions are designed, whether they explicitly account for the progressive nature of dementia, and how they adapt to shifting caregiver responsibilities and challenges over time. By mapping the characteristics of these interventions, we aim to identify gaps in phase-specific support and highlight opportunities for enhancing caregiver-centered intervention design. This is particularly relevant given that dementia caregiving is a dynamic process, requiring tailored and timely support that responds to caregivers’ changing emotional, practical, and informational needs. A key contribution of this review is its focus on phase-specific intervention delivery. While many caregiving programs exist, their effectiveness in addressing caregivers’ evolving needs remains unclear. Some interventions may be effective in the early stages of dementia but fail to support caregivers as the disease progresses. Understanding whether existing interventions adjust their content, intensity, and mode of delivery based on these caregiving phases is crucial for optimizing support for family caregivers. Future directions for research will be identified as part of the discussion of the results of this review. Particularly, our results could help researchers and program administrators better understand how to tailor their interventions to different phases of the dementia caregiving trajectory. This scoping review will be part of a larger program of research aimed at exploring the impact of time-based support on family caregivers to persons to dementia on caregiving efficacy and health and social outcomes. We believe that this review could be a major step towards informing future interventions to better meet the evolving needs of family caregivers to persons with dementia.

### Limitations

This scoping review has several limitations that should be acknowledged. First, by restricting our inclusion criteria to trials, we may exclude valuable insights from qualitative and mixed-methods studies that provide a deeper understanding of caregivers’ lived experiences and the contextual factors influencing intervention effectiveness. While RCTs offer robust evidence on efficacy, they may not fully capture the real-world applicability and feasibility of interventions across diverse settings. Although we will include translated studies where feasible, some relevant interventions in other languages may not be captured. Third, this review examines dementia caregiving interventions spanning different stages of the illness trajectory; however, variability in how studies define and categorize these stages may pose challenges for comparison and synthesis. The heterogeneity of interventions, settings, and outcome measures across included studies may further limit our ability to draw definitive conclusions regarding intervention effectiveness at different caregiving stages. Finally, while we will conduct a methodological appraisal of included RCTs, the absence of a formal meta-analysis limits our ability to quantitatively assess intervention effectiveness. As a scoping review, our aim is to map the existing literature rather than determine intervention efficacy, and future systematic reviews with meta-analyses may be needed to address this gap.

## Conclusion

We present a protocol for a forthcoming scoping review that will provide a critical synthesis of dementia caregiving interventions, evaluating their alignment with the evolving needs of family caregivers across the disease trajectory. By mapping interventions onto the CIP-AD framework, we will highlight whether existing support programs are structured to adapt to caregivers’ changing emotional, informational, and practical challenges [11]. Our findings will contribute to a deeper understanding of how caregiving interventions are designed and delivered, identifying gaps in phase-specific support and areas for future research. The implications of our review extend beyond academic inquiry. We expect that insights gained from our review will inform policymakers, healthcare providers, and program developers in structuring more responsive and dynamic support systems for caregivers. Rather than a one-size-fits-all approach, interventions should be tailored to meet caregivers’ evolving needs, ensuring that they receive timely and appropriate support throughout their journey. Our engagement with knowledge users ensures that the insights gained will be relevant to caregivers, healthcare providers, and organizations supporting dementia care, ultimately contributing to more tailored and effective caregiving interventions.

## Supporting information

S1 File Prisma-P checklist. (PDF)
